# Genome Sequence of Jumbo Phage vB_AbaM_ME3 of *Acinetobacter baumanni*

**DOI:** 10.1128/genomeA.00431-16

**Published:** 2016-08-25

**Authors:** Colin Buttimer, Lisa O’Sullivan, Mohamed Elbreki, Horst Neve, Olivia McAuliffe, R. Paul Ross, Colin Hill, Jim O’Mahony, Aidan Coffey

**Affiliations:** a Department of Biological Sciences, Cork Institute of Technology, Co. Cork, Ireland; b Department of Microbiology and Biotechnology, Max Rubner-Institut, Kiel, Germany; c Biotechnology Department, Teagasc, Moorepark Food Research Centre, Fermoy, Co. Cork, Ireland; d Department of Microbiology, University College Cork, Co. Cork, Ireland

## Abstract

Bacteriophage (phage) vB_AbaM_ME3 was previously isolated from wastewater effluent using the propagating host *Acinetobacter baumannii* DSM 30007. The full genome was sequenced, revealing it to be the largest *Acinetobacter* bacteriophage sequenced to date with a size of 234,900 bp and containing 326 open reading frames (ORFs).

## GENOME ANNOUNCEMENT

*Acinetobacter baumannii* has emerged in recent times as an important nosocomial pathogen. Health care-acquired *A. baumannii* infections include pneumonia and urinary tract and bloodstream infections (1). There is only a small number of bacteriophages (phages) with genomes greater than 200 kbp (termed “jumbo” phages) that have had their genomes sequenced to date. Most of their encoded proteins do not have any homologues in current sequence databases, and the diversity of these phages has been great enough that it has limited comparative genomics studies (2).

A phage with the ability to lyse *A. baumannii* strain DSM 30007 was isolated from effluent obtained from a wastewater treatment plant in Cork, Ireland. Transmission electron microscopy revealed that the phage belonged to the *Myoviridae* family, and according to nomenclature proposed by Kropinski et al. was named vB_AbaM_ME3 (ME3) (3). A high titer phage suspension was concentrated by ultracentrifugation, and DNA extraction was performed as previously described (4). DNA was sequenced using the 454 FLX Titanium PLUS Sequencing approach (LGC Genomics, Mannheim, Germany). Open reading frames (ORFs) were identified using GLIMMER and GenemarkS (5, 6), with possible function of these ORFs’ proteins being predicted with BLASTp, pFam, InterProScan, THMHMM v.2.0, LipoP v1.0 (7–11), with tRNAscan.SE 1.21 being used to locate any tRNA present in the genome (12).

To date, this is the largest *Acinetobacter* phage genome sequenced, with a size of 234,900 bp (the genome ends of ME3 are not known). The overall %G+C is 40%, similar to that of its host (13). The genome was predicted to have 326 ORFs with four tRNA genes.

On the basis of homology, putative functions were assigned to 77 ORFs, with 19 ORFs annotated as putative membrane proteins, two ORFs annotated as putative lipoproteins, and the remaining 228 ORFs being annotated as hypothetical proteins.

Phage ME3 is an orphan phage, however, it has eight ORFs encoding structural proteins that share homology to those of the novel *Bacillus* phage 0305phi8-36 (GenBank accession number NC_009760.1), although showing high levels of divergence (percentage identity of 26% to 34%). The major head protein (ME3_22), portal protein (ME3_19), and tail sheath subunit (ME3_29) are examples of such proteins. Until now, these proteins of 0305phi8-36 have only been found to share homology with those of phage-like elements found in the genomes of *B. thuringiensis* serovar *israelensis* and *B. weihenstephanensis* (14). With regard to these structural proteins and the large terminase subunit (ME3_13), phages ME3 and 0305phi8-36 may share a distant ancestor.

Phage ME3 appears to encode its own DNA replication machinery including DNA polymerase subunits (ME3_60 and 61), thymidylate synthase enzymes (ME3_107 and 108), helicases, and enzymes involved in DNA degradation and repair. ME3 also possesses two cell wall degrading enzymes, ME3_8, a lysozyme with proven lytic activity against *A. baumannii* and ME3_113, a putative cell wall hydrolase. Phage ME3 also has a curiously large number of genes associated with Ter-stress response (ME3_286, 284, 289, 290, and 291) and a massive protein of 5,419 amino acids (ME3_104) possessing domains relating to host specificity and binding (IPR015406, IPR008979).

### Accession number(s).

The full genome sequence of *A. baumannii* phage vB_AbaM_ME3 was deposited in GenBank under the accession number KU935715.
